# Differences in knee-ankle configuration and ankle joint kinetic characteristics during a drop landing vertical jump task in highly trained male basketball players: a cross-sectional study based on rebound efficiency

**DOI:** 10.3389/fpsyg.2026.1904882

**Published:** 2026-07-30

**Authors:** Yanzhi He, Zilong Wang, Wenxin Sun, Huizi Cui, Bojun Zhou, Jianming Wu, Jianfu Cheng, Chaofu Chen

**Affiliations:** 1 School of Physical Education, Jimei University, Xiamen, China; 2 Sports Science Research Institute, Jimei University, Xiamen, China

**Keywords:** ankle joint moment, basketball players, foot-ankle biomechanics, jumping, knee-ankle configuration, rebound efficiency

## Abstract

**Objective:**

This study compared contact-phase timing, knee–ankle configuration at the lowest center of mass (COM), and ankle-moment characteristics between basketball players with high and low drop landing vertical jump (DLVJ) rebound efficiency.

**Methods:**

Forty-one highly trained male basketball players were classified as high (*n* = 21) or low efficiency (*n* = 20). Participants performed a 30-cm box DLVJ while three-dimensional kinematic and kinetic data were recorded. Between-group differences were tested using two-sided Mann–Whitney U tests with Benjamini–Hochberg false discovery rate (FDR) correction. Spearman rank correlations between rebound efficiency and biomechanical variables were examined in the full sample.

**Results:**

The high-efficiency group achieved greater DLVJ height (*p* = 0.014, q = 0.027) and rebound efficiency (*p* < 0.001, q < 0.001), whereas relative squat one-repetition maximum did not differ (*p* = 0.235, q = 0.353). Contact, braking, and propulsive times were shorter in the high-efficiency group (all *p* < 0.001, all q < 0.001). At the lowest COM, knee flexion and ankle dorsiflexion angles were smaller (*p* < 0.001, q < 0.001; *p* = 0.006, q = 0.016, respectively). The high-efficiency group also showed a greater mean ankle moment during braking (*p* = 0.014, q = 0.027) and reached peak ankle moment earlier during braking (*p* = 0.002, q = 0.007) and propulsion (*p* = 0.007, q = 0.016). Peak ankle moment during braking and propulsion showed uncorrected between-group differences (both *p* = 0.046), but neither remained significant after FDR correction (both q = 0.079).

**Conclusion:**

Greater DLVJ rebound efficiency in highly trained basketball players was associated with shorter contact-phase durations, smaller knee flexion and ankle dorsiflexion at the lowest COM, and earlier ankle-moment timing during landing-to-repropulsion. These differences were not accompanied by higher relative maximal squat strength, suggesting that rebound efficiency reflects task-specific landing-transition characteristics rather than maximal lower-limb strength alone. Because contact time is included in the rebound-efficiency calculation, temporal findings should be interpreted as component characteristics, not independent outcomes. Knee–ankle configuration and ankle-moment timing may offer task-specific observational information on distal ankle-joint kinetic involvement during DLVJ performance.

## 1 Introduction

Basketball is a multidimensional team sport characterized by frequent high-intensity intermittent actions. During training and competition, basketball players repeatedly execute explosive movement transitions, including acceleration, change of direction, jumping, and landing, thereby imposing substantial demands on sport-specific physical capacity and neuromuscular control (Stojanovic et al., 2018; Ben Abdelkrim et al., 2010; Harper et al., 2019). In this context, the physical capacities and movement strategies of highly trained athletes tend to be more sport-specific; consequently, biomechanical analyses conducted in highly trained basketball players may provide a more ecologically relevant understanding of the actual control demands imposed by basketball-specific tasks (McKay et al., 2022; Ben Abdelkrim et al., 2010). Time-motion analyses have shown that basketball players frequently perform jumping and landing tasks during competition. Elite under-19 male basketball players complete approximately 44 jumps per game, whereas semiprofessional male basketball players may perform approximately 146 jumps per game, most of which involve bilateral take-off patterns (Abdelkrim et al., 2007; Talpey et al., 2021). Repeated jumping and landing expose the lower limbs to substantial mechanical loading within short time intervals; peak vertical ground reaction force during bilateral landing has been reported to reach approximately 2.38–4.91 body weights, underscoring the mechanical demands of landing attenuation and subsequent repropulsion (Niu et al., 2014). During such explosive actions, players are typically required to complete braking, center-of-mass redirection, and subsequent propulsion within a brief contact window, which is often shorter than the time required to develop absolute maximal force. Thus, rapid force production and temporal organization are particularly important for basketball-specific jumping performance (Tillin and Bishop, 2009; Flanagan and Comyns, 2008). Although vertical jump height differs meaningfully across competitive levels, and male basketball players have been reported to display vertical jump heights of approximately 40–75 cm, basketball-specific jump performance should not be interpreted solely on the basis of jump height; rather, contact time, braking-to-propulsion transition, and the capacity for repropulsion after landing should also be considered (Ziv and Lidor, 2010; Miura et al., 2010; Sugiyama et al., 2014). Therefore, analyzing temporal efficiency and phase-specific biomechanics during a landing-to-take-off task may provide a more specific evaluation of lower-limb functional performance in basketball players and may contribute to the refinement of physical performance assessment (Flanagan and Comyns, 2008; Abdelkrim et al., 2007; Talpey et al., 2021).

Within this sport-specific context, the biomechanical characteristics of highly trained basketball players are likely to have stronger task representativeness. Basketball players of different training and competitive levels may differ in anthropometry, lower-limb neuromuscular performance, and sport-specific explosive capacity; these differences may be reflected in age category, competitive standard, playing position, and vertical jump force-time characteristics associated with on-court contribution (Ben Abdelkrim et al., 2010; Cabarkapa et al., 2024; Philipp et al., 2023). Compared with developmental or lower-level athletes, high-level basketball players typically demonstrate greater vertical jumping ability and higher force-power outputs, indicating more pronounced sport-specific adaptations in explosive force production (Cabarkapa et al., 2024; Philipp et al., 2023; Ziv and Lidor, 2010). Such functional advantages are generally underpinned by long-term systematic training and a high degree of movement proficiency, which may strengthen the interpretive relevance of associations among strength capacity, neuromuscular control, and sport-specific performance in high-level athletes (Ben Abdelkrim et al., 2010; McKay et al., 2022; Suchomel et al., 2016). For high-intensity basketball actions such as vertical jumping, acceleration, and rapid change of direction, maximal lower-limb strength represents an important foundation for rapid force production and explosive performance, whereas relative strength may more accurately capture the capacity to overcome body mass during sport-specific movement tasks (Benítez-Flores et al., 2024; Suchomel et al., 2016; Suchomel et al., 2018). Nevertheless, basketball-specific jumping performance depends not only on jump height but also on the ability to brake rapidly after landing, redirect the center of mass, and repropel the body into take-off; therefore, temporal organization and lower-limb control during landing-to-repropulsion should also be considered (Flanagan and Comyns, 2008; Miura et al., 2010; Xu et al., 2024b). Accordingly, focusing on highly trained basketball players may facilitate a more precise understanding of the relationship between drop landing vertical jump (DLVJ) rebound efficiency and the control characteristics of impact attenuation, rapid transition, and repropulsion under a relatively stable sport-specific training background (Ben Abdelkrim et al., 2010; McKay et al., 2022; Xu et al., 2024a).

The DLVJ integrates impact absorption after dropping from a specified height, contact-phase braking, and subsequent vertical repropulsion, and may therefore serve as a laboratory-based representation of the ability to execute a second take-off after landing in basketball. In the present study, DLVJ rebound efficiency was defined as DLVJ height (m) divided by total contact time (s). This metric integrates the vertical displacement achieved after landing with the total time spent in contact during the task (Flanagan and Comyns, 2008; Xu et al., 2023; Xu et al., 2024b). Compared with a simple vertical jump, the DLVJ incorporates jump height, contact time, braking time, propulsive time, and pre-take-off repropulsive ability, making it well suited to characterizing the landing-to-propulsion transition in a fast stretch-shortening cycle task (Flanagan and Comyns, 2008; Xu et al., 2024b; Xu et al., 2023). Research on basketball-specific jumping likewise suggests that jump performance should not be interpreted exclusively from jump height, because contact time, lower-limb kinematic behavior, and rapid repropulsive capacity are also important components of basketball-specific jumping ability (Miura et al., 2010; Sugiyama et al., 2014). Thus, analyzing the DLVJ in highly trained basketball players may reveal temporal organization and lower-limb control characteristics during impact absorption, transition at the lowest center of mass, and subsequent repropulsion (Flanagan and Comyns, 2008; Xu et al., 2023; Xu et al., 2024b). In the landing-to-repropulsion process represented by the DLVJ, the foot-ankle complex constitutes the distal mechanical interface through which the body interacts with the ground. Its range of motion and moment generation contribute to impact attenuation and subsequent propulsion (Hertel and Corbett, 2019; Simpson et al., 2019). Ankle injuries are commonly reported in basketball, and rebounding and other jump-landing actions have been identified as common contexts for their occurrence (Morris et al., 2021; Lempke et al., 2021; Tummala et al., 2018). In addition to their clinical relevance, ankle-joint motion and moment generation are mechanically important for controlling the interaction between the lower limb and the ground during rapid landing-to-repropulsion tasks. Previous research on basketball-specific jumping and rebound-jump tasks suggests that ankle-joint angle and moment timing may provide useful descriptors of foot-ankle behavior during landing and subsequent take-off (Wang et al., 2023; Xu et al., 2023; Yoshida et al., 2024). However, it remains unclear whether different levels of DLVJ rebound efficiency in highly trained basketball players are accompanied by differences in contact-phase timing, sagittal-plane knee-ankle configuration at the lowest center of mass, and sagittal-plane ankle moment magnitude and timing. Therefore, examining knee-ankle configuration at the lowest center of mass, peak ankle moment, mean ankle moment, and the timing of peak ankle moment during the DLVJ may help elucidate distal ankle-joint kinetic involvement in basketball players during landing-to-repropulsion.

Therefore, this study primarily compared contact-phase temporal characteristics, sagittal-plane knee-ankle configuration at the lowest center of mass, and sagittal-plane ankle joint kinetic characteristics between basketball players with high and low DLVJ rebound efficiency during a rapid landing-to-repropulsion task. The analysis was restricted to task-specific sagittal-plane variables and was intended to describe ankle-joint kinetic involvement during DLVJ performance. Complementary continuous analyses were also conducted to examine associations between DLVJ rebound efficiency and all available biomechanical variables across the full sample. It was hypothesized that, compared with the low-rebound-efficiency group, the high-rebound-efficiency group would exhibit shorter contact-phase durations, a distinct sagittal-plane knee-ankle configuration at the lowest center of mass, and earlier sagittal-plane ankle moment timing.

## 2 Participants and methods

### 2.1 Participants

A sensitivity analysis for statistical power was conducted using G*Power software (version 3.1.9.2; Heinrich-Heine-Universität Düsseldorf, Germany) for the comparison between the high- and low-DLVJ-rebound-efficiency groups. Based on the final sample size (21 participants in the high-rebound-efficiency group and 20 participants in the low-rebound-efficiency group), the test family was specified as “t tests,” the statistical test as “Means: Difference between two independent means (two groups),” and the type of power analysis as “Sensitivity: Compute required effect size - given *α*, power, and sample size.” Under a two-sided test, α = 0.05, statistical power = 0.80, and an allocation ratio of 20/21, the present study had approximately 80% power to detect a between-group difference of Cohen’s d ≈ 0.90. In previous jump-landing studies, absolute Cohen’s d values for kinematic variables related to lateral ankle ligament injury in elite athletes have ranged from approximately 0.05 to 1.68, whereas those for ankle variables during the basketball three-step layup have ranged from approximately 0.13 to 2.12 (Lin et al., 2019; Wang et al., 2023). According to Cohen’s commonly used benchmarks, d = 0.80 may be interpreted as a large effect (Cohen, 1992). Accordingly, the present study was primarily powered to detect large between-group effects, and may have been underpowered for small-to-moderate effects.

A total of 41 highly trained male basketball-specialist participants were included in the final analysis. Participants had 7.7 ± 1.3 years of systematic basketball training experience and had completed at least 2 years of structured strength and conditioning training. The sample comprised guards and forwards in approximately equal proportions, with no centers. Based on the participant classification framework and the documented basketball-specific training background, the sample was positioned between Tier 2 and Tier 3, corresponding to trained to highly trained athletes (McKay et al., 2022).

Because no established clinical or performance cut-off exists for DLVJ rebound efficiency, participants were ranked according to their DLVJ rebound-efficiency values and classified using the sample median of 0.9008325057. Participants with rebound-efficiency values greater than or equal to the sample median were assigned to the high-rebound-efficiency group (*n* = 21), whereas those with values below the median were assigned to the low-rebound-efficiency group (*n* = 20). The single participant with a value equal to the median was assigned to the high-rebound-efficiency group according to this prespecified rule. This median-based classification was used to compare biomechanical profiles across relatively high and low levels of DLVJ rebound efficiency and was not intended as a clinical diagnostic threshold or a training-level classification criterion (Deng et al., 2025; Xu et al., 2024a).

### 2.2 Inclusion and exclusion criteria

The inclusion criteria were as follows: (1) age between 18 and 25 years; (2) systematic basketball training experience and at least two years of structured strength and conditioning training; (3) proficiency in the barbell back squat and jump-landing tasks, as confirmed by a strength and conditioning coach; and (4) ability to complete the 30-cm box DLVJ task under standardized conditions. Barbell back squat 1RM and relative squat 1RM were measured to characterize the maximal strength background of the high- and low-DLVJ-rebound-efficiency groups.

The exclusion criteria were as follows: (1) severe lower-limb bone, muscle, ligament, or neurological injury within the preceding six months that required medical intervention or led to interruption of systematic training; (2) acute lower-limb pain or evident movement limitation during testing; (3) concurrent participation in other physical training interventions that could affect the study outcomes; and (4) failure to complete the DLVJ task according to the required criteria or missing key kinematic or kinetic data.

### 2.3 Instrumentation

**Figure 1:**
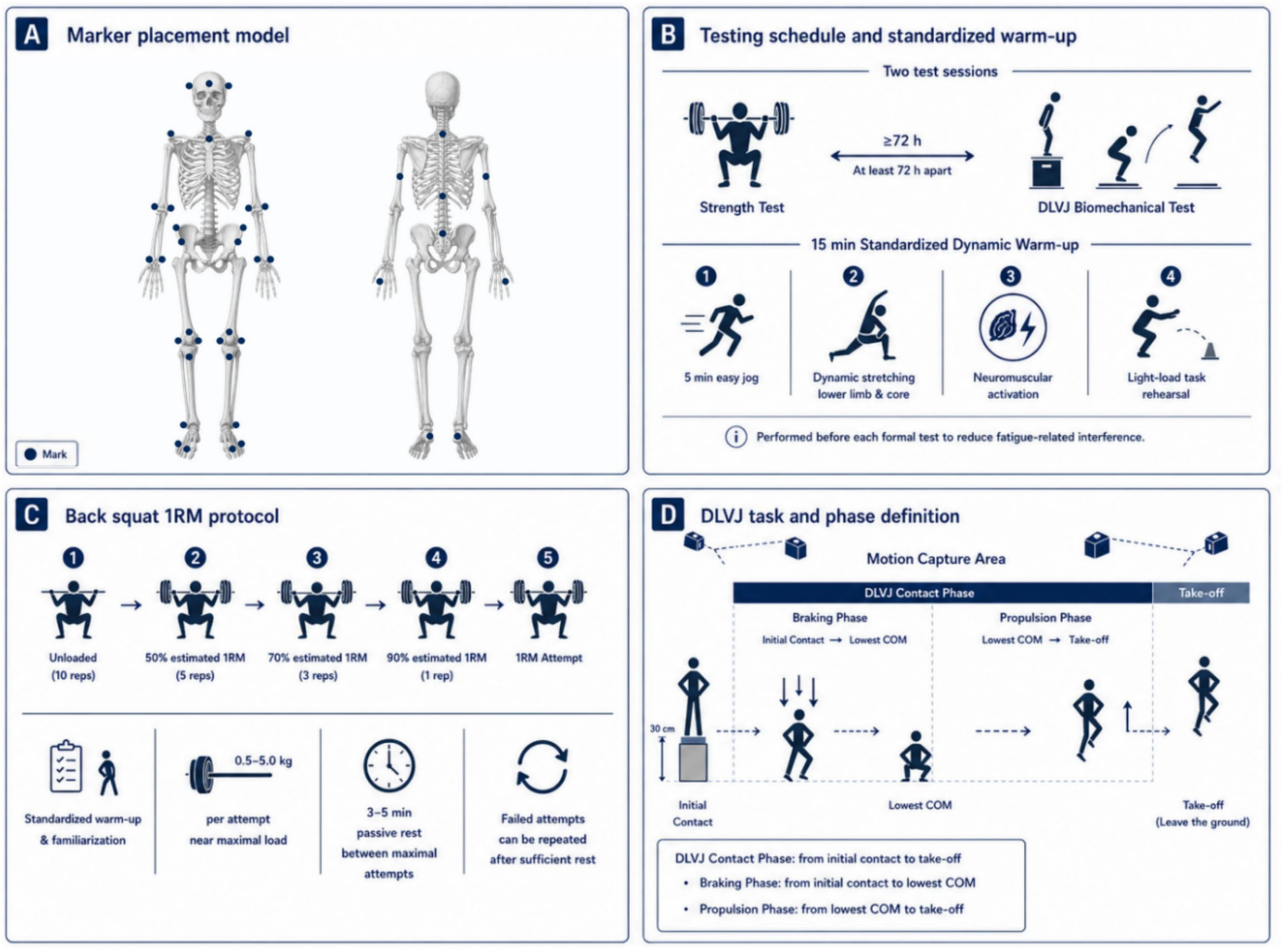
Schematic illustration of the experimental design and testing procedures. **(A)** Reflective marker placement model; **(B)** Testing schedule and standardized warm-up; **(C)** Barbell back squat 1RM testing protocol; **(D)** DLVJ task and contact-phase segmentation.

Three-dimensional kinematic data were collected at 200 Hz using a 14-camera infrared motion-capture system (4 Arqus and 10 Miqus cameras; Qualisys AB, Gothenburg, Sweden). Reflective markers were placed on anatomical landmarks according to the Qualisys functional assessment marker set and were used to construct a CAST six-degrees-of-freedom rigid-body model (Figure 1A). The foot, ankle, knee, and hip anatomical landmarks, rigid-body model, and joint-angle definitions were implemented according to this marker set and the CAST model. Synchronized ground reaction force data were collected at 1000 Hz using two embedded three-dimensional piezoelectric force plates (Type 9287CA; Kistler Group, Winterthur, Switzerland). The motion-capture system and force plates were synchronized through Qualisys Track Manager software to ensure temporal alignment between kinematic and kinetic data.

### 2.4 Experimental procedures

The study comprised two testing sessions: strength testing and DLVJ biomechanical testing. The two sessions were separated by at least 72 h to minimize the influence of neuromuscular fatigue on subsequent performance. Before each formal testing session, participants completed a standardized 15-min dynamic warm-up consisting of 5 min of low-intensity jogging, dynamic stretching of the lower limbs and core, neuromuscular activation exercises, and light-load task-specific rehearsal (Figure 1B).

#### 2.4.1 Barbell Back squat 1RM testing

After the first-stage warm-up, participants completed barbell back squat 1RM testing. The 1RM test is a commonly used method for assessing maximal strength and has acceptable reliability when conducted with standardized warm-up, familiarization, and sufficient rest (Seo et al., 2012; Grgic et al., 2020). Participants first performed approximately 10 familiarization repetitions with an unloaded bar. They then completed 50% of the estimated 1RM for 5 repetitions, 70% for 3 repetitions, and 90% for 1 repetition. During near-maximal attempts, the load was increased by 0.5 to 5.0 kg per attempt until a valid 1RM was obtained. Passive rest periods of 3 to 5 min were provided between maximal attempts, and failed attempts could be repeated after sufficient rest (Figure 1C).

#### 2.4.2 Drop landing vertical jump (DLVJ) testing

DLVJ task. In the present study, DLVJ referred specifically to a bilateral drop landing vertical jump in which participants stepped naturally forward and downward from a 30-cm box, landed with each foot on a separate force plate, and immediately performed a maximal-effort vertical jump. Participants maintained their hands on their hips to minimize the influence of arm swing on jump performance and lower-limb kinetics, consistent with standardized jump-testing procedures (Bobbert et al., 1987; Anicic et al., 2023). Participants were not permitted to actively jump upward from the box. Although the DLVJ shares a drop-and-immediate-repropulsion structure with conventional drop-jump and rebound-jump protocols, the present task differed from conventional drop-jump protocols that prioritize minimal contact time. Participants were instructed to maximize jump height after landing rather than minimize contact time. This task structure is consistent with the broader use of landing-to-repropulsion tasks to characterize fast stretch-shortening cycle performance while preserving a task instruction distinct from conventional short-contact-time drop-jump testing (Bobbert et al., 1987; Flanagan and Comyns, 2008; Xu et al., 2024b). Each participant completed at least three valid DLVJ trials with sufficient rest between attempts, and the valid trial with the greatest jump height was selected for analysis (Figure 1D). Because the task instruction prioritized maximal jump height, this prespecified selection was used to characterize the contact time and sagittal-plane lower-limb biomechanics accompanying each participant’s maximal height-oriented DLVJ performance. Contact time was not used as a trial-selection criterion; rather, it was interpreted as the temporal characteristic associated with the trial in which maximal jump height was achieved. All kinematic and kinetic variables were extracted from the same trial to preserve the correspondence between the performance outcome and its associated biomechanical characteristics. As verbal instructions can influence time-dependent and kinetic characteristics of jump performance, the present findings should be interpreted within this height-oriented task instruction (Sánchez-Sixto et al., 2024).

### 2.5 Data acquisition and processing

Data processing was performed using Visual3D software (version 2021a; C-Motion, Inc., Germantown, MD, USA). Kinematic trajectories and ground reaction force data were filtered using a fourth-order zero-lag Butterworth low-pass filter with a cut-off frequency of 15 Hz; this parameter is explicitly reported because low-pass filtering can influence inverse-dynamics estimates of joint moments (Kristianslund et al., 2012). The two force plates recorded left- and right-side ground reaction forces separately, and DLVJ event detection was based on the combined vertical ground reaction force signal from both force plates. Foot contact with an individual force plate was identified using a 10 N vertical ground reaction force threshold. Initial contact was defined as the instant at which either force plate exceeded 10 N and the combined vertical ground reaction force indicated the onset of contact; take-off was defined as the instant at which both force plates fell below 10 N and the combined vertical ground reaction force returned to the no-contact state (Wang et al., 2025; Wang et al., 2026b). The DLVJ contact phase was defined from initial contact to take-off; the braking phase was defined from initial contact to the lowest center of mass; and the propulsive phase was defined from the lowest center of mass to take-off (Bobbert et al., 1987; Flanagan and Comyns, 2008; Xu et al., 2024b). The lowest COM was selected as the primary kinematic event because it marks the reversal of the vertical COM trajectory from descent to ascent and simultaneously delineates the braking and propulsive phases in the present DLVJ framework. It therefore provided a common, task-relevant reference point for comparing sagittal-plane joint configuration at the braking-to-propulsion transition; it was not intended to represent the peak excursion of each individual joint. Contact time, braking time, and propulsive time were calculated from the corresponding event timings (Wang et al., 2026a).

### 2.6 Statistical analysis

All statistical analyses were conducted using Python (version 3.10). Continuous variables are presented as mean ± standard deviation (SD), and distributional characteristics were assessed using the Shapiro–Wilk test together with visual inspection. Between-group differences between the high- and low-DLVJ-rebound-efficiency groups were examined using two-sided Mann–Whitney U tests, and effect sizes were expressed as Cliff’s *δ*. The direction of Cliff’s δ was defined as the high-rebound-efficiency group relative to the low-rebound-efficiency group; negative values indicate lower values in the high-rebound-efficiency group, whereas positive values indicate higher values in the high-rebound-efficiency group. Cliff’s *δ* reflects the difference between the probability that a randomly selected value from the high-rebound-efficiency group exceeds a randomly selected value from the low-rebound-efficiency group and the reverse probability (Cliff, 1993). Effect-size magnitudes were interpreted as negligible (|δ| < 0.147), small (0.147 ≤ |δ| < 0.330), medium (0.330 ≤ |δ| < 0.474), or large (|δ| ≥ 0.474) (Romano et al., 2006). To complement the group-based comparison, two-sided Spearman rank correlation analyses were conducted between DLVJ rebound efficiency and 16 prespecified performance and biomechanical variables in the full sample. The continuous analyses included DLVJ height; contact, braking, and propulsive time; sagittal-plane hip, knee, and ankle angles at the lowest center of mass; sagittal-plane ankle moment variables; and hip and knee joint-moment background variables. The exploratory four-group strength-rebound-efficiency analysis was examined using the Kruskal-Wallis test. When the omnibus test reached statistical significance, Dunn *post hoc* comparisons were performed with corresponding multiple-comparison adjustment. The Benjamini-Hochberg procedure was applied separately to the between-group comparisons, the 16 continuous correlations, the four-group Kruskal-Wallis analyses, and the Dunn post hoc comparisons. Statistical significance was set at *α* = 0.05.

## 3 Results

### 3.1 Participant characteristics and DLVJ performance

A total of 41 highly trained collegiate basketball-specialist participants were included in the final analysis, comprising 21 participants in the high-rebound-efficiency group and 20 participants in the low-rebound-efficiency group. Age, height, body mass, squat 1RM, and relative squat 1RM did not differ statistically between groups (all *p* > 0.05, all FDR-corrected q > 0.05), indicating comparable basic anthropometric and maximal-strength backgrounds. Compared with the low-rebound-efficiency group, the high-rebound-efficiency group showed greater DLVJ height (*p* = 0.014, q = 0.027, Cliff’s *δ* = 0.452) and greater DLVJ rebound efficiency (*p* < 0.001, q < 0.001, Cliff’s δ = 1.000). Table 1 also presents the between-group comparisons for phase-time variables, knee-ankle configuration at the lowest center of mass (COM), and ankle joint kinetic variables, providing an integrated numerical basis for subsequent interpretation (Table 1).

**Table 1:** Participant characteristics, DLVJ performance, and primary biomechanical variables in the high- and low-DLVJ-rebound-efficiency groups.

Variable	High-rebound-efficiency group *n*	High-rebound-efficiency group Mean ± *SD*	Low-rebound-efficiency group *n*	Low-rebound-efficiency group Mean ± *SD*	*p* value; FDR *q* value	Cliff’s *δ*
Age (years)	21	19.95 ± 0.86	20	19.70 ± 0.80	0.274; 0.366	0.190
Height (cm)	21	181.43 ± 5.25	20	181.10 ± 5.51	0.814; 0.858	0.045
Body mass (kg)	21	72.67 ± 7.93	20	75.80 ± 8.63	0.261; 0.366	−0.207
Squat 1RM (kg)	21	125.71 ± 16.98	20	127.50 ± 19.50	0.822; 0.858	−0.043
Relative squat 1RM (kg/kg)	21	1.74 ± 0.19	20	1.69 ± 0.21	0.235; 0.353	0.219
DLVJ height (cm)	21	46.96 ± 5.54	20	43.79 ± 3.26	0.014; 0.027	0.452
DLVJ rebound efficiency	21	1.17 ± 0.40	20	0.76 ± 0.08	<0.001; <0.001	1.000
DLVJ contact time (s)	21	0.43 ± 0.08	20	0.59 ± 0.05	<0.001; <0.001	−0.929
DLVJ braking time (s)	21	0.20 ± 0.04	20	0.28 ± 0.03	<0.001; <0.001	−0.914
DLVJ propulsive time (s)	21	0.23 ± 0.04	20	0.30 ± 0.03	<0.001; <0.001	−0.840
Knee angle at lowest COM (°)	21	103.26 ± 14.61	20	121.56 ± 24.14	<0.001; <0.001	−0.700
Ankle dorsiflexion angle at lowest COM (°)	21	33.52 ± 5.47	20	38.32 ± 8.99	0.006; 0.016	−0.500
Peak ankle moment during braking (N·m·kg^−1^)	21	2.52 ± 1.16	20	1.98 ± 1.58	0.046; 0.079	0.367
Peak ankle moment during propulsion (N·m·kg^−1^)	21	2.95 ± 1.23	20	2.47 ± 1.63	0.046; 0.079	0.367
Mean ankle moment during braking (N·m·kg^−1^)	21	1.35 ± 0.72	20	0.91 ± 0.76	0.014; 0.027	0.452
Time to peak ankle moment during braking (s)	21	0.18 ± 0.06	20	0.23 ± 0.09	0.002; 0.007	−0.560
Time to peak ankle moment during propulsion (s)	21	0.09 ± 0.06	20	0.16 ± 0.09	0.007; 0.016	−0.498

DLVJ, drop landing vertical jump; COM, center of mass; 1RM, one-repetition maximum; SD, standard deviation. Between-group comparisons were performed using two-sided Mann–Whitney U tests. FDR q values were calculated using the Benjamini-Hochberg method, treating the comparisons between the high- and low-rebound-efficiency groups as a single statistical family. The direction of Cliff’s δ is defined as the high-rebound-efficiency group relative to the low-rebound-efficiency group; positive values indicate higher values in the high-rebound-efficiency group, whereas negative values indicate lower values in the high-rebound-efficiency group. Hip, knee, and ankle angles were defined as 0° in the static upright posture; positive values indicate hip and knee flexion and ankle dorsiflexion. Joint moments were normalized to body mass and are reported in N·m·kg^−1^.

### 3.2 DLVJ phase-time characteristics and knee-ankle joint configuration at the lowest center of mass

**Figure 2:**
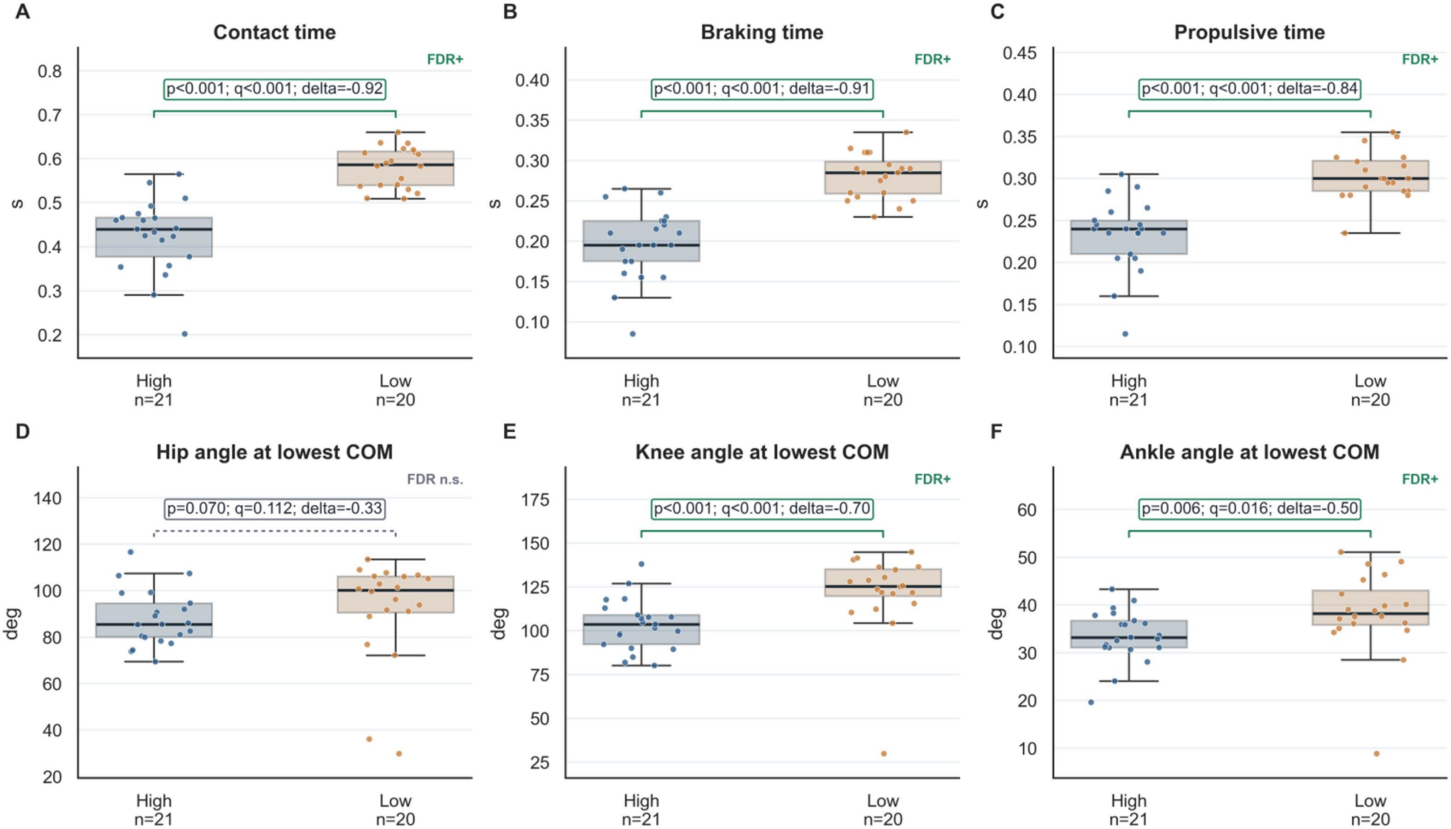
Phase-time characteristics and lower-limb joint angles at the lowest center of mass in the high- and low-DLVJ-rebound-efficiency groups: contact time **(A)**; braking time **(B)**; propulsive time **(C)**; hip angle at the lowest center of mass **(D)**; knee angle at the lowest center of mass **(E)**; ankle angle at the lowest center of mass **(F)**. Box plots show the interquartile range and median, individual dots represent participant data, and each panel reports raw *p*, Benjamini-Hochberg FDR *q*, and Cliff’s delta.

The high-rebound-efficiency group exhibited shorter DLVJ phase durations. Compared with the low-rebound-efficiency group, the high-rebound-efficiency group had shorter contact time (*p* < 0.001, q < 0.001, Cliff’s *δ* = −0.929), shorter braking time (*p* < 0.001, q < 0.001, Cliff’s *δ* = −0.914), and shorter propulsive time (*p* < 0.001, q < 0.001, Cliff’s δ = −0.840). The high-rebound-efficiency group also displayed smaller knee angles and smaller ankle dorsiflexion angles at the lowest center of mass. Specifically, the knee angle at the lowest center of mass was lower in the high-rebound-efficiency group than in the low-rebound-efficiency group (*p* < 0.001, q < 0.001, Cliff’s δ = −0.700), and the ankle dorsiflexion angle was also lower (*p* = 0.006, q = 0.016, Cliff’s δ = −0.500). The between-group difference in hip angle at the lowest center of mass did not reach statistical significance (*p* = 0.070, q = 0.112, Cliff’s δ = −0.333) (Figure 2).

### 3.3 Ankle joint kinetic characteristics and hip-knee kinetic background variables

**Figure 3:**
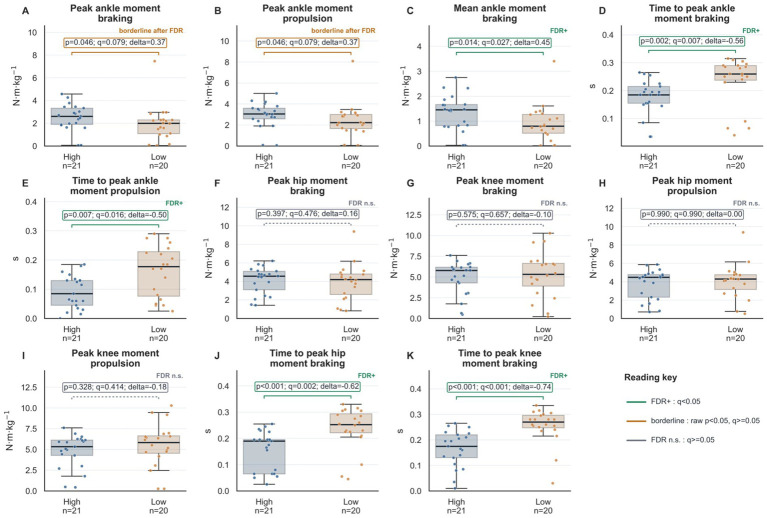
Joint moment characteristics in the high- and low-DLVJ-rebound-efficiency groups. Panel **(A,B)** show peak ankle moment magnitudes during braking and propulsion; panel (C) Shows mean ankle moment during braking; panel **(D,E)** show time to peak ankle moment; panel **(F–I)** show hip and knee peak moment magnitudes; panel **(J,K)** show time to peak hip and knee moment during braking. Green labels indicate FDR-significant results, grey labels indicate non-significant results, and amber labels indicate raw *p* < 0.05 findings that did not remain significant after FDR correction.

The high-rebound-efficiency group demonstrated earlier ankle joint moment timing and a greater mean ankle moment during braking. Compared with the low-rebound-efficiency group, the high-rebound-efficiency group exhibited a greater mean ankle moment during braking (*p* = 0.014, q = 0.027, Cliff’s δ = 0.452). The high-rebound-efficiency group reached peak ankle moment earlier during braking (*p* = 0.002, q = 0.007, Cliff’s δ = −0.560) and propulsion (*p* = 0.007, q = 0.016, Cliff’s δ = −0.498). The uncorrected *p* values for peak ankle moment during braking and propulsion were both 0.046; however, these differences did not remain statistically significant after FDR correction (both q = 0.079). Therefore, the peak ankle moment magnitude results should be interpreted as borderline differences. The magnitudes of peak hip and knee moments did not differ statistically between groups, although the timing of peak moments during braking occurred earlier in the high-rebound-efficiency group. Peak hip moment during braking (*p* = 0.397, q = 0.476), peak knee moment during braking (*p* = 0.575, q = 0.657), peak hip moment during propulsion (*p* = 0.990, q = 0.990), and peak knee moment during propulsion (*p* = 0.328, q = 0.414) did not differ statistically between groups. In contrast, the high-rebound-efficiency group reached peak hip moment earlier during braking (*p* < 0.001, q = 0.002, Cliff’s δ = −0.619) and also reached peak knee moment earlier during braking (*p* < 0.001, q < 0.001, Cliff’s δ = −0.736) (Figure 3).

### 3.4 Exploratory strength-rebound-efficiency classification analysis

**Figure 4:**
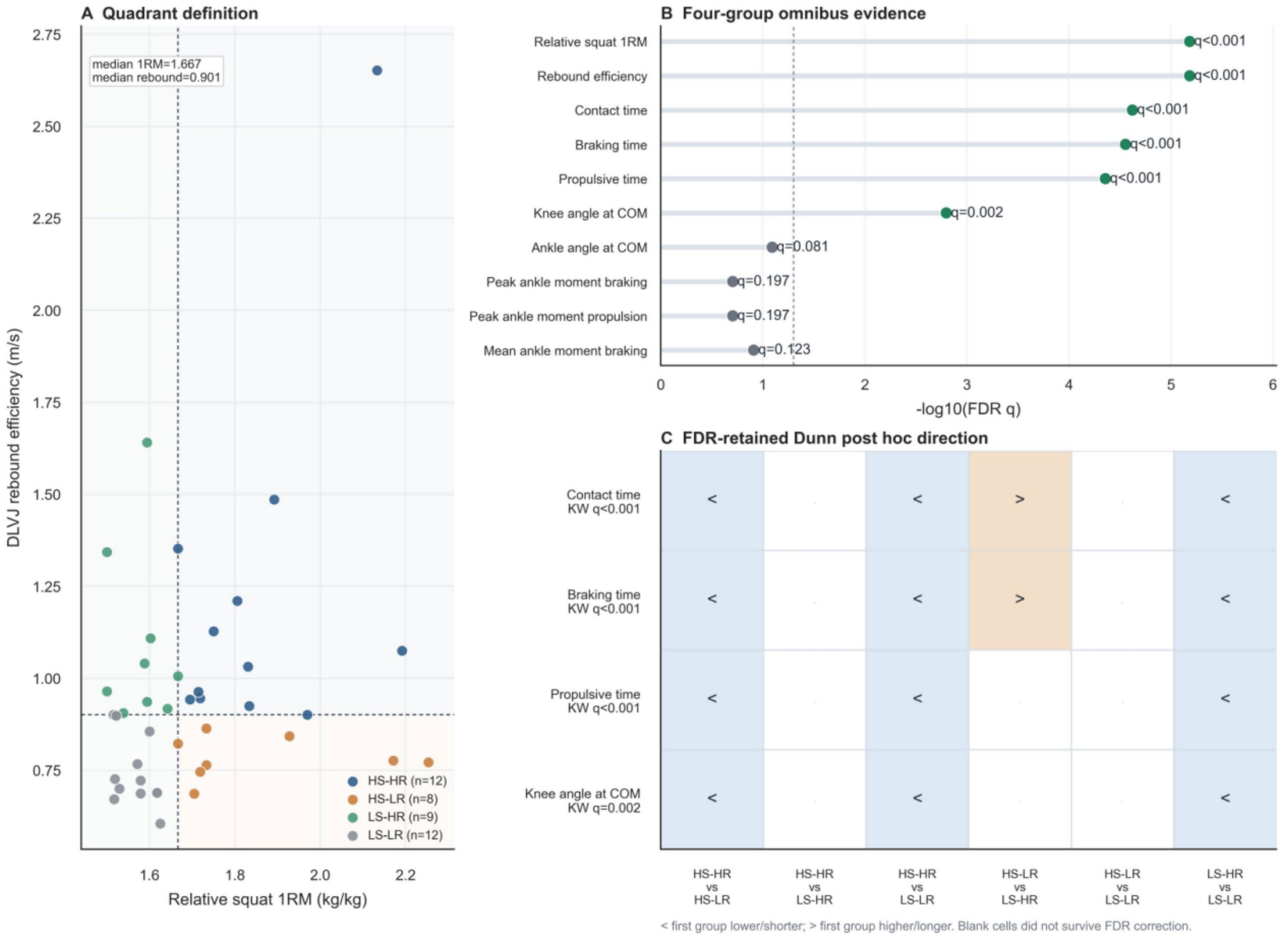
Exploratory four-group classification based on relative squat strength and DLVJ rebound efficiency. Panel **(A)** shows the distribution of participants across four quadrants defined by the median values of relative squat 1RM and DLVJ rebound efficiency. Panel **(B)** presents the FDR-corrected Kruskal–Wallis omnibus *q* values across outcome variables. Panel **(C)** summarizes the direction of Dunn *post hoc* comparisons that remained statistically significant after FDR correction.

The exploratory strength-rebound-efficiency classification was used primarily to describe combined patterns of maximal strength background and rebound efficiency. Participants were categorized into a high-strength/high-rebound-efficiency group (HS-HR, *n* = 12), a high-strength/low-rebound-efficiency group (HS-LR, *n* = 8), a low-strength/high-rebound-efficiency group (LS-HR, *n* = 9), and a low-strength/low-rebound-efficiency group (LS-LR, *n* = 12). As classification-defining variables, relative squat 1RM and DLVJ rebound efficiency differed statistically across the four groups (Kruskal-Wallis H = 30.108 and 30.438, respectively; both *p* < 0.001, both q < 0.001); these results were used only to verify the classification and were not interpreted as independent findings. Among interpretable task outcomes, four-group differences were observed for contact time (H = 26.597, *p* < 0.001, q < 0.001), braking time (H = 25.677, *p* < 0.001, q < 0.001), propulsive time (H = 24.269, *p* < 0.001, q < 0.001), and knee angle at the lowest center of mass (H = 16.365, *p* < 0.001, q = 0.002). FDR-corrected Dunn *post hoc* comparisons showed that the HS-HR group had shorter contact time, braking time, and propulsive time than both the HS-LR and LS-LR groups; the LS-HR group had shorter values for all three phase-time variables than the LS-LR group; and the HS-LR group had longer contact time and braking time than the LS-HR group. For knee angle at the lowest center of mass, the HS-HR group showed lower values than the HS-LR and LS-LR groups, and the LS-HR group showed lower values than the LS-LR group. The overall four-group difference in ankle dorsiflexion angle at the lowest center of mass did not remain statistically significant after FDR correction (H = 7.536, *p* = 0.057, q = 0.081). Peak ankle moment during braking, peak ankle moment during propulsion, and mean ankle moment during braking did not differ statistically among the four groups after FDR correction (H = 4.678, 4.688, and 6.292, respectively; *p* = 0.197, 0.196, and 0.098; q = 0.197, 0.197, and 0.123) (Figure 4).

### 3.5 Continuous associations between DLVJ rebound efficiency and biomechanical variables

Rebound efficiency was positively associated with DLVJ height (Spearman’s *ρ* = 0.592, *p* < 0.001, q < 0.001) and inversely associated with contact time (ρ = −0.900, *p* < 0.001, q < 0.001), braking time (ρ = −0.872, *p* < 0.001, q < 0.001), and propulsive time (ρ = −0.841, *p* < 0.001, q < 0.001). Because rebound efficiency is calculated from jump height and total contact time, and braking plus propulsive time equals total contact time, these associations were treated as descriptive component relationships rather than independent biomechanical findings. Among variables not directly included in the rebound-efficiency calculation, greater rebound efficiency was associated with smaller hip flexion angle (ρ = −0.373, *p* = 0.016, q = 0.022), knee flexion angle (ρ = −0.641, *p* < 0.001, q < 0.001), and ankle dorsiflexion angle (ρ = −0.398, *p* = 0.010, q = 0.014) at the lowest center of mass. Greater rebound efficiency was also associated with a greater peak ankle plantarflexion moment during braking (ρ = 0.349, *p* = 0.025, q = 0.031), a greater mean ankle plantarflexion moment during braking (ρ = 0.425, *p* = 0.006, q = 0.009), earlier peak hip moment timing during braking (ρ = −0.610, *p* < 0.001, q < 0.001), earlier peak knee moment timing during braking (ρ = −0.687, *p* < 0.001, q < 0.001), earlier peak ankle moment timing during braking (ρ = −0.502, *p* < 0.001, q = 0.002), and earlier peak ankle moment timing during propulsion (ρ = −0.494, *p* = 0.001, q = 0.002). Peak ankle moment during propulsion and peak hip and knee moments during braking were not significantly associated with rebound efficiency after FDR correction (all q ≥ 0.059) (Table 2).

**Table 2:** Spearman rank correlations between DLVJ rebound efficiency and prespecified performance and biomechanical variables (*n* = 41).

Variable	Spearman’s *ρ*	*p* value	FDR q value
DLVJ height	0.592	<0.001	<0.001
DLVJ contact time	−0.900	<0.001	<0.001
DLVJ braking time	−0.872	<0.001	<0.001
DLVJ propulsive time	−0.841	<0.001	<0.001
Hip flexion angle at lowest COM	−0.373	0.016	0.022
Knee flexion angle at lowest COM	−0.641	<0.001	<0.001
Ankle dorsiflexion angle at lowest COM	−0.398	0.010	0.014
Peak ankle moment during braking	0.349	0.025	0.031
Peak ankle moment during propulsion	0.306	0.052	0.059
Mean ankle moment during braking	0.425	0.006	0.009
Time to peak hip moment during braking	−0.610	<0.001	<0.001
Time to peak knee moment during braking	−0.687	<0.001	<0.001
Time to peak ankle moment during braking	−0.502	<0.001	0.002
Time to peak ankle moment during propulsion	−0.494	0.001	0.002
Peak hip moment during braking	0.103	0.520	0.555
Peak knee moment during braking	−0.046	0.775	0.775

DLVJ, drop landing vertical jump; COM, center of mass. Two-sided Spearman rank correlations were calculated in the full sample. FDR q values were calculated using the Benjamini-Hochberg procedure across the 16 prespecified continuous correlations.

## 4 Discussion

This study compared contact-phase temporal characteristics, sagittal-plane knee-ankle configuration at the lowest center of mass, and sagittal-plane ankle joint kinetic characteristics between highly trained male basketball players with high and low DLVJ rebound efficiency. The DLVJ task incorporates landing impact absorption, braking-to-propulsion transition, and subsequent repropulsive take-off and therefore enables the description of lower-limb behavior during rapid redirection of the center of mass after landing. Overall, the findings partially supported the predefined hypotheses. The high-rebound-efficiency group demonstrated smaller knee flexion and ankle dorsiflexion angles at the lowest center of mass, a greater mean ankle plantarflexion moment during braking, and earlier peak ankle moment timing during braking and propulsion. Complementary continuous analyses showed that rebound efficiency was associated with selected sagittal-plane kinematic and kinetic variables across the full sample. It should be noted that although the uncorrected *p* values for peak ankle moment magnitude during braking and propulsion reached the 0.05 level, these differences did not remain statistically significant after FDR correction and should therefore be interpreted with caution. Specifically, basketball players with high DLVJ rebound efficiency displayed faster phase transitions, smaller knee angles and ankle dorsiflexion angles at the lowest center of mass, a greater mean ankle moment during braking, and earlier peak ankle moment timing during the rapid landing-to-take-off task. These findings suggest that the high-rebound-efficiency group was able to complete post-landing braking, transition, and repropulsion more rapidly during the DLVJ. This characteristic is unlikely to be explained solely by between-group differences in maximal lower-limb strength and may instead relate to temporal organization, knee-ankle geometry, and distal foot-ankle kinetic involvement under short-contact conditions. This interpretation is consistent with the emphasis placed by Flanagan and Comyns on contact time and reactive-strength indices in fast stretch-shortening cycle tasks, and with Komi’s classical theory of elastic energy storage and rapid force production (Flanagan and Comyns, 2008; Komi, 2000). In addition, the work of Xu et al. (2023) on joint work and landing-to-repropulsion characteristics during rebound-jump tasks supports the view that the DLVJ reflects not only jump height but also lower-limb temporal organization and kinetic coordination during the contact phase. In the context of the frequent jump-landing demands of basketball, the present findings provide task-specific information on sagittal-plane ankle-joint kinetic involvement during landing-to-repropulsion. However, because ankle-sprain mechanisms are inherently multiplanar and frontal-plane ankle mechanics were not assessed, these findings should not be interpreted as evidence of ankle-sprain mechanisms, injury risk, or injury-prevention effects.

The phase-time results were directionally consistent with Hypothesis 1, whereas the ankle kinetic results partially supported Hypothesis 3. The high-rebound-efficiency group exhibited shorter contact time, braking time, and propulsive time than the low-rebound-efficiency group. This pattern describes a shorter temporal profile across the contact phase of the DLVJ task. The phase-time findings should, however, be interpreted in relation to the computational basis of DLVJ rebound efficiency, because this metric is jointly determined by jump height and total contact time. Accordingly, the shorter contact time observed in the high-rebound-efficiency group was partly inherent to the grouping metric, whereas braking time and propulsive time represented component phases of total contact time. Flanagan and Comyns proposed that contact time and reactive-strength indices are important variables for evaluating fast stretch-shortening cycle performance, and Xu et al. similarly emphasized that contact time and take-off performance jointly contribute to rebound-jump performance (Flanagan and Comyns, 2008; Xu et al., 2023; Xu et al., 2024a). Within this framework, the concurrent shortening of braking and propulsive time provides additional descriptive information regarding the distribution of total contact time across the landing-to-repropulsion task. These findings do not establish that the high-rebound-efficiency group completed impact attenuation, transition at the lowest center of mass, or repropulsive take-off more effectively. They also do not directly demonstrate a superior temporal strategy, greater elastic-energy utilization, or more efficient stretch-shortening cycle function. Komi’s conceptualization of rapid eccentric-concentric transition and the findings of Yoshida et al. regarding lower-limb kinetic variables in drop-jump performance provide theoretical context for interpreting rapid landing-to-repropulsion tasks, yet direct verification of these mechanisms requires measures of joint work, musculotendinous behavior, stiffness, and muscle activation (Komi, 2000; Yoshida et al., 2024). Corresponding to this phase-time profile, the high-rebound-efficiency group exhibited a greater mean sagittal-plane ankle plantarflexion moment during braking and reached peak ankle moment earlier during braking and propulsion. Peak ankle moment magnitudes during braking and propulsion did not remain statistically significant after FDR correction and therefore should not be emphasized as robust primary differences. The present results more strongly support the interpretation that between-group differences were concentrated in ankle moment timing and mean ankle moment during braking, rather than in a generalized increase in ankle peak moment magnitude or proximal joint peak moments. The complementary continuous analyses showed a similar pattern, with greater rebound efficiency associated with a greater mean ankle plantarflexion moment during braking and earlier peak ankle moment timing during braking and propulsion. Previous studies have identified ankle joint kinetic and timing variables as relevant descriptors of lower-limb behavior during basketball-specific jump-landing and rebound-jump tasks (Wang et al., 2023; Xu et al., 2023; Yoshida et al., 2024). In the present study, earlier attainment of peak ankle moment indicates that the peak net internal ankle plantarflexion moment occurred earlier within the corresponding braking or propulsive phase. This temporal characteristic may be relevant to the description of ankle kinetic organisation during the DLVJ task. It should not be interpreted as direct evidence of a greater capacity for rapid force production, faster neuromuscular activation, or superior distal-joint control, because rate of force development, electromyographic activity, and tissue-specific mechanical behavior were not measured. Therefore, the present findings support an association between DLVJ rebound efficiency and selected sagittal-plane ankle kinetic characteristics during the specific landing-to-repropulsion task, without establishing causal or mechanistic relationships.

The knee-ankle configuration findings supported Hypothesis 2. The high-rebound-efficiency group demonstrated smaller sagittal-plane knee flexion and ankle dorsiflexion angles at the lowest center of mass, whereas hip flexion angle did not differ statistically between groups. The complementary continuous analyses were generally consistent with the group-based findings, showing that greater DLVJ rebound efficiency was associated with smaller hip, knee, and ankle angles at the lowest center of mass. Taken together, these findings indicate that the observed differences were concentrated primarily in the sagittal-plane knee-ankle configuration at the transition from braking to propulsion, rather than reflecting a uniform alteration across all lower-limb joints. The present findings should, however, be interpreted cautiously. Smaller knee flexion and ankle dorsiflexion angles at the lowest center of mass describe the joint configuration adopted during the DLVJ task, but they do not, by themselves, establish the mechanical or neuromuscular mechanisms underlying that configuration. In particular, the present study did not quantify vertical stiffness, leg stiffness, ankle joint stiffness, joint work, musculotendinous behavior, or muscle activation. Accordingly, the observed angular pattern should not be interpreted as direct evidence of greater foot-ankle stiffness, greater lower-limb stiffness, enhanced elastic-energy storage and return, or superior neuromuscular control. Existing biomechanical frameworks indicate that lower-limb stiffness should be quantified directly from force-displacement relationships, whereas joint stiffness requires direct evaluation from joint moment-angle relationships; neither construct can be inferred reliably from discrete joint angles alone (Butler et al., 2003; Serpell et al., 2012). Nevertheless, the observed configuration may provide a useful task-specific description of how players with different DLVJ rebound-efficiency levels organize the sagittal-plane knee-ankle system during rapid landing-to-repropulsion. Previous work has shown that landing configuration and movement strategy can influence reactive-strength-related performance characteristics during jumping tasks, although the magnitude and functional interpretation of these relationships are dependent on task instructions, drop height, participant characteristics, and the definition of performance outcomes (Guy-Cherry et al., 2018; Flanagan and Comyns, 2008; Xu et al., 2024b). This consideration is particularly relevant in the present study because participants were instructed to maximize jump height after landing rather than minimize contact time, and the DLVJ should therefore not be considered equivalent to conventional short-contact-time drop-jump protocols. The observed knee-ankle configurations should not be interpreted as injury-risk patterns because the present analysis was restricted to sagittal-plane variables at the lowest center of mass during a bilateral DLVJ task. Direct comparison with injury-related landing patterns or inference regarding multiplanar ankle-sprain mechanisms is therefore not warranted. A conservative interpretation is that higher DLVJ rebound efficiency co-occurred with a more extended sagittal-plane knee-ankle configuration at the lowest center of mass during this specific landing-to-repropulsion task. Whether this configuration is related to stiffness regulation, joint work redistribution, muscle activation strategy, foot-ankle functional capacity, or other neuromuscular mechanisms remains an untested hypothesis. Future studies should directly quantify vertical, leg, and ankle stiffness using prespecified force-displacement and moment-angle approaches, and should integrate phase-specific joint work, electromyographic activity, foot-ankle functional measures, and repeated-trial analyses to clarify the mechanisms underlying these configurational differences (Butler et al., 2003; Serpell et al., 2012; Wang et al., 2023). Evidence from another weight-bearing task indicates that knee joint loading can vary with squat depth and movement speed, reinforcing that joint-angle-to-load relations are task dependent and cannot be transferred directly to the present DLVJ (Li W. et al., 2025).

The relative squat 1RM and exploratory classification results constrain the interpretation of maximal strength background and practical application in this study. Relative squat 1RM did not differ statistically between groups, indicating that the shorter phase durations, earlier ankle moment timing, and greater mean ankle moment during braking in the high-rebound-efficiency group cannot be attributed simply to greater maximal squat strength. Grgic et al. (2020) and Seo et al. (2012) have both noted that 1RM testing is a commonly used and reliable method for assessing maximal strength; however, the present findings further suggest that, when maximal strength background is comparable, DLVJ rebound efficiency may more strongly reflect temporal organization, knee-ankle coordination, and foot-ankle kinetic responses during rapid landing-to-repropulsion, rather than a single advantage in maximal strength. This interpretation is consistent with studies by Xu et al. on the DLVJ and rebound-jump performance, which indicate that rebound-jump tasks are influenced not only by strength level but also by contact time, joint kinetics, and phase-specific control strategies (Xu et al., 2024a). The exploratory strength-rebound-efficiency classification showed marked differences in phase-time variables across profiles, whereas peak ankle moment variables were not stable. This suggests that maximal strength background and rebound efficiency may occur in different combinations; however, this analysis should be treated as a supplementary description and should not replace the primary comparison between high- and low-DLVJ-rebound-efficiency groups. From a measurement perspective, the DLVJ may be used to characterize jump height, contact time, sagittal-plane knee-ankle configuration at the lowest center of mass, and sagittal-plane ankle moment timing during a standardized landing-to-repropulsion task. Such a multi-variable assessment may provide information beyond that obtained from isolated jump-height or maximal-strength measures, because jump height and contact-time performance represent related but non-identical qualities in basketball players (Miura et al., 2010; Sugiyama et al., 2014). However, cross-sectional studies measure variables at a single point in time and cannot establish temporal sequence or causality (Wang and Cheng, 2020). Accordingly, the present results cannot determine whether sagittal-plane knee-ankle configuration, ankle moment timing, or other observed characteristics are modifiable through training; whether they cause differences in DLVJ rebound efficiency; or whether they predict ankle-injury outcomes. The findings should therefore be interpreted as task-specific observational information that may inform performance monitoring and hypothesis generation. Future prospective and randomized intervention studies should examine whether targeted changes in landing-to-repropulsion coordination, knee-ankle movement organization, or rapid moment-development characteristics modify DLVJ rebound efficiency and biomechanical variables not mathematically included in its calculation. Before any injury-related application is considered, prospective studies should incorporate frontal-plane ankle mechanics and injury-surveillance data; the present variables should not be treated as standalone injury-risk criteria or clinical screening thresholds.

This study has four main limitations. First, the cross-sectional design precludes causal inference and cannot determine whether the observed biomechanical characteristics precede, result from, or can be modified to influence DLVJ rebound efficiency. In addition, the sample was restricted to highly trained male collegiate basketball-specialist participants; therefore, the findings may not generalize to female athletes, athletes of other competitive levels, or athletes from other sports. Second, the biomechanical assessment was limited to sagittal-plane joint angles at the lowest center of mass and sagittal-plane ankle moment variables derived from the dominant limb. Frontal-plane ankle mechanics, complete contact-phase waveforms, bilateral coordination, electromyography, phase-specific joint work, ankle range of motion, and direct measures of joint or leg stiffness were not assessed. Accordingly, the present findings cannot characterize multiplanar ankle loading or be extrapolated to ankle-sprain mechanisms, injury risk, or injury-prevention effects. Third, DLVJ rebound efficiency is mathematically derived from jump height and contact time. Therefore, associations between rebound efficiency and contact-phase time variables should be interpreted as component relationships rather than independent biomechanical findings. In addition, the sample-median dichotomization discarded within-group variation, reduced statistical efficiency, and produced group membership that depended on the distribution of the present cohort. Although the continuous correlation analyses retained information across the full sample, they did not eliminate the structural dependency between rebound efficiency and its component variables or convert the sample median into a generalizable threshold. Fourth, the primary analysis was based on the valid trial with the greatest jump height because the task instruction emphasized maximal jump height. This approach was intended to characterize the contact time and lower-limb biomechanics accompanying maximal height-oriented DLVJ performance rather than average behavior across repeated trials; however, it may not fully represent each participant’s typical movement strategy. Because trial means and within-session reliability were not reported, future studies should examine repeated-trial reliability and determine whether the present findings remain consistent when biomechanical variables are averaged across standardized valid trials.

## 5 Conclusion

In highly trained basketball players, greater DLVJ rebound efficiency was associated with shorter contact-phase durations, a specific knee-ankle configuration at the lowest center of mass, a greater mean ankle moment during braking, and earlier ankle moment timing during the rapid landing-to-repropulsion process, rather than simply indicating superior maximal lower-limb strength. Knee-ankle configuration at the lowest center of mass and ankle moment magnitude and timing during the DLVJ may provide task-specific information for understanding landing-transition characteristics and distal ankle-joint kinetic involvement in basketball players. These findings should be considered observational and may inform future prospective and randomized intervention studies rather than causal training or injury-prevention recommendations.

## Data Availability

The raw data supporting the conclusions of this article will be made available by the authors, without undue reservation.
